# Seroprevalence and risk factors of *Borrelia burgdorferi* sensu lato and *Rickettsia* species infection in humans in Mongolia, 2016–2020

**DOI:** 10.1371/journal.pone.0289274

**Published:** 2023-08-08

**Authors:** Dashdavaa Ganbold, Bayarsaikhan Uudus, Naranbat Nyamdavaa, Yeruult Chultemsuren, Amarbayasgalan Zagd, Mungunzaya Tangad, Agarzandan Bayarmaa, Rolomjav Lkunrev, Uyanga Baasandagva, Tsogbadrakh Nyamdorj, Myadagsuren Narankhajid

**Affiliations:** 1 Department of Biology, School of Biomedicine, Mongolian National University of Medical Sciences, Ulaanbaatar, Mongolia; 2 Department of Biology, School of Sciences and Art Science, National University of Mongolia, Ulaanbaatar, Mongolia; 3 Gyals Medical Centre, Ulaanbaatar, Mongolia; 4 National Centre for Zoonotic Diseases, Ulaanbaatar, Mongolia; National Veterinary Research Institute (NVRI), NIGERIA

## Abstract

*Borrelia burgdorferi* sensu lato and *Rickettsia* spp. are worldwide causes of tick-borne infections. We aimed to estimate the seroprevalence of immunoglobulin G (IgG) antibodies against different tick-borne diseases (TBDs) and determine risk factors among Mongolians from 2016 to 2020. Blood samples were obtained from voluntary participants with a history of suspected tick bite who visited our hospital, and IgG antibodies against *Rickettsia* and *Borrelia* were detected using enzyme-linked immunosorbent assay (ELISA). The IgG antibody seropositivity rate against *Rickettsia* was 21.8% (1032/4724), while 3.4% (162/4724) of participants tested positive for serum IgG antibodies against *Borrelia* by ELISA.Binary logistic regression analysis was performed to evaluate risk factors for tick-borne rickettsiosis (TBR) and tick-borne borreliosis (TBB) using IgG serum sample. Age, occupation, and residence were significantly associated with these diseases; however, sex did not show any significant association. Seroprevalence was significantly higher among herders (40.6%, 95% confidence interval [CI]: 35.5–45.8; odds ratio [OR] 0.61; *P* < 0.001) and students (32.8%, 95% CI: 30.2–35.4; OR 0.75; *P* < 0.001) than among individuals with other occupations. The 25–29 age group had a slightly higher seroprevalence (35.1%, 95% CI: 28.1–42.6; OR 0.61; *P* < 0.006) than those in other age groups. Province was a stronger predictor of TBR than occupation and age group. In univariate subgroup analysis by age group, occupation, and residence were significantly associated with TBR seroprevalence, whereas age and province were associated with TBB seroprevalence. Thus, risk factors for TBD include residence, occupation, and age group. This study was conducted using samples from all Mongolian provinces and the capital city, and the risk factors and prevalence of *Rickettsia* and *Borrelia*are highlighted.

## Introduction

Tick-borne diseases (TBDs) are a growing public health concern in Mongolia and a cause of significant disease burden in humans because ticks serve as vectors in the transmission of pathogens [1–3]. Tick-borne borreliosis (TBB; also known as Lyme disease) is caused by the spirochete *Borrelia burgdorferi* sensu lato, whereas tick-borne rickettsiosis (TBR) is caused by a gram-negative intracellular bacterium; TBB and TBR constitute TBDs worldwide, with clinical manifestations [1].Tick-borne borreliosis causes several neurological and arthritic symptoms, such as headache, paralysis, and erythema migrans [2]. Tick-borne rickettsiosis usually manifests as mild fever, muscle ache, rash, cough, and nausea [3].Several studies have evaluated the seroprevalence of *Borrelia* and*Rickettsia* in herders [3,4], blood donors in Mongolia [5], forestry workers in various Europeancountries [6–8], farmers in Poland [6], and blood donors in California [9]; however, no large-scale study has comprehensively investigated this aspect in all Mongolian provinces, while differentiating between different occupations.

Seroprevalence surveys can provide valuable data regarding the epidemiology and diagnosis of TBDs. First, as antibodies persist in the blood for years after symptomatic and asymptomatic infection, seropositivity indicates the cumulative occurrence of infection in individuals. Second, knowledge of the baseline seroprevalence is crucial for appropriate interpretation of antibody test results in terms of their predictive diagnostic value [9].Serum antibody detection constitutes the standard method for diagnosing TBB and TBR [7,9,10]. Immunoglobulin M (IgM) istypically used as a marker for the serodiagnosis of current infection, while immunoglobulin G (IgG) is used to identify recent infection.However, diagnosis is complicated by several factors; in the early stages of infection (up to 8 weeks), antibodies may be absent, whereas antibodies may persist for years after recovery from disease [10].Therefore, to have therapeutic value, the clinical presentation and background seroprevalence should be considered in interpreting serological test results[11].

The seroprevalence of IgG antibodies against *B*. *burgdorferi* sensu lato and *Rickettsia* has been evaluated in different countries [3,4,6,7,9]. In blood donors in southern Norway, the seroprevalence of serum IgG antibodies against *Borrelia* and *Rickettsia* was 22% and 4.2%, respectively [10].The seroprevalence of *Borrelia* ranged from 20% to 62% in forestry workers in Europe [7], 18.2% to 50.7% in Polish farmers [6], 5% to 23% in Italian forestry workers, and 14% to 20% in French forestry workers [7].The global seroprevalence of *B*. *burgdorferi* is estimated at 14.5%, and the three regions with the highest seroprevalence are Central Europe (20.7%), East Asia (15.9%), and Western Europe (13.5%) [12,13].

In previous Mongolian studies that assessed the seroprevalence of IgG antibodies against *B*. *burgdorferi* sensu lato, the seroprevalence was 1.9% in the Selenge and Bulgan Provinces, 3% in the Tuv Province, 13.9% in the Dornogobi Province, and 25% in the Zavkhan Province [5,14]. The seroprevalence of IgG antibodies against *Rickettsia* spp. was 17.5% in herders, 19.5% in non-herders, and 20.4% in livestock [3,4].

In Mongolia, *Borrelia* and *Rickettsia*have been detected in ticks [1,15],humans [3–5,14], and small mammals [16]. Determining the human seroprevalence of IgG antibodies against TBDs plays a vital role in clinical diagnosis and facilitates the enhancement of public health awareness [12]. Therefore, this study was conducted to estimate the seroprevalence of TBB and TBR to inform public health approaches and future research on *Borrelia* and *Rickettsia*.

## Materials and methods

### Study area

Mongolia is located in north-central Asia, bordered by Russia in the north and China in the south, and is a landlocked mountainous country with an area of 1, 564,116 square kilometres (603,909 square miles). Its average altitude is 1,580 m (5,180 ft) above sea level. Mongolia’s geography is very diverse, and the country is often classified under six ecological areas: highland, mountain-taiga, forest-steppe, steppe, desert-steppe, and desert. Mongolia consists of 21 administrative provinces with a population of approximately 3.3 million. In 2021, the population density was 2.5 people/km^2^. Approximately 60% of the population lives in Ulaanbaatar, the capital of Mongolia, and nearly 40% of the rural population are nomadic herders [17].

### Study population

This study was conducted between 2016 and 2020. Samples were collected from the general hospital in each province and sent to the National Centre for Zoonotic Diseases for analysis. The study examined individuals in the provinces that seek and access healthcare services. Whole blood was collected from patients with symptoms of fever, headache, rash, eschar, and lymphadenopathy after a suspected tick bite (S1–S7 Files).

### Enzyme-linked immunosorbent assays

All samples were collected from every general hospital in each province, and the whole blood was allowed to clot for l–2 h at room temperature, and then serum was separated in tubesafter incubating at 20°C for 15 min and centrifuging at 1200 g.Samples were then sent to the main laboratory of the National Centre for Zoonotic Diseases, Ulaanbaatar, Mongolia. Enzyme-linked immunosorbent assay (ELISA) was performed to detect *Borrelia* spp. (*B*. *afzelii*, *B*. *garinii*, and *B*. *burgdorferi* sensu stricto) using an Anti-*Borrelia* Plus VlsE ELISA for IgG kit (Euroimmun AG, Aktiengesellschaft, Lübeck, Germany) (dx.doi.org/10.17504/protocols.io.kxygx9j7kg8j/v1S8). All serum samples were tested for *Rickettsia* IgG using a commercial ELISA kit (NovaTec Immunodiagnostica GmbH, Dietzenbach, Germany) according to the manufacturer’s instructions (dx.doi.org/10.17504/protocols.io.bp2l69jdrlqe/v1S9). The optical density (OD) of the ELISA plates was read using an ELISA plate reader (Infinite F50, Tecan). OD values were measured at 450 and 620 nm according to the manufacturer’s instructions. The results were calculated in NovaTec units (NTUs) as the mean absorbance value multiplied by 10 and divided by the mean cut-off value. Antibody indices were calculated by dividing the OD of the test sample by the average OD of the cut-off calibrators (provided with positive and negative serum discrimination kits). The NTU values were determined according to the manufacturer’s instructions. Samples with NTU <8 were considered negative, while those with NTU >11 were considered positive. We analysed 4724 patients’ sera for *Rickettsia* and *Borrelia*. Samples with NTU values between 9 and 11 were considered ambiguous, and the test was repeated. If the results remained ambiguous, the sample was considered to test negative.

### Informed consent and questionnaire

The purpose of the study, study procedures, and the informed consent form were explained to patients in detail.Parents or guardians and potential participants were allowed to ask questions.

After obtaining informed consent, participants were required to answer a printed version of the study questionnaire, which included questions regarding demographics, age, sex, exposure to tick bites, previous tick-borne infections, and other diagnoses. If the participants were younger than 18 years, their parents or guardians provided the information in the questionnaire and consent to participate; participants aged 18–75 years filled the questionnaire themselves. A researcher assisted illiterate participants in filling the questionnaire (S1–S7 Files).

### Data analysis

In this study, all data were analysed using R version 4.0.3 (The R Foundation for Statistical Computing, Vienna, Austria) [18].Binary logistic regression models were used to calculate seropositivity in the study groups. The results were reported as odds ratios (ORs) with 95% confidence intervals (CIs). Statistical significance was set at *P* < 0.05. Generalised additive models were used to determine age trend.

#### Risk factor analysis

The seroprevalence of *Rickettsia* antibodies was associated with age; therefore, age group and study year were included in the risk factor analysis. The crude and adjusted ORs were estimated for age, sex, occupation, and province of residence.

### Ethics

The study was conducted in accordance with the Declaration of Helsinki and was approved by the Medical Ethical Committee of the Mongolian National University of Medical Sciences (MEC No. 18-02/2A). Written informed permission was obtained from all participants (or parents/guardians for participants younger than 18 years).

## Results

### Study group

Whole-blood samples were obtained from 4724 participants (0–75 years), of whom 37.2% (1758/4724) were male and 62.8% (2966/4724) were female.

### Seroprevalence of tick-borne rickettsiosis

**Fig 1:**
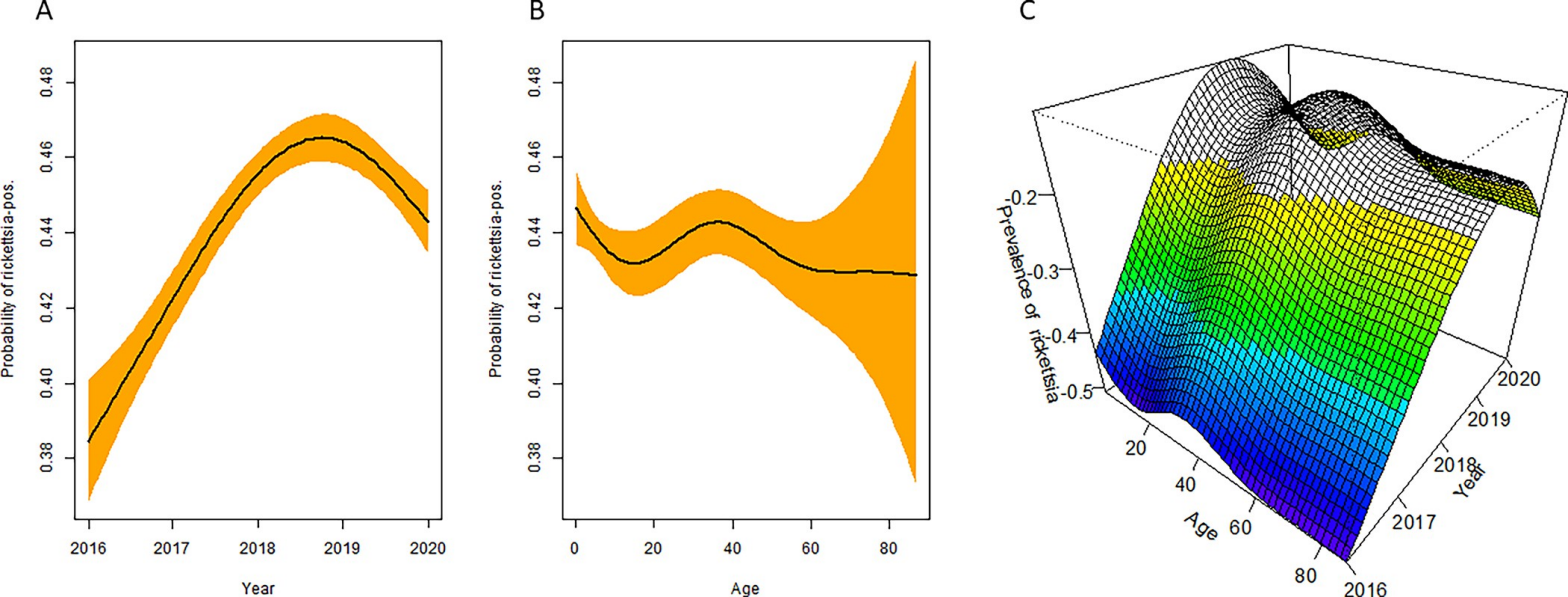
Overall *Rickettsia* seroprevalence trends by age. *Rickettsia* seroprevalence is plotted based on generalised additive model trend curves, with 95% confidence intervals assessed from cluster bootstrapping 1000 by year (A) and age (B). Three-dimensional age-specific contour maps for *Rickettsia* seroprevalence probability for the target year (C).

The seroprevalence of *Rickettsia* was 29.2% (95% CI: 27.4–31.2%) and 25.8% (95% CI: 23.6–28.1%) in female and male participants, respectively (Table 1). From 2016 to 2020, the TBR seroprevalence showed a curve and differed significantly by year (*P* < 0.001), with a peak of 46% in 2018 (Fig 1A).

**Table 1:** Stratified seroprevalence of IgG antibodies against *Rickettsia* spp. detected by ELISA in participants aged 0–75 years and the results of binary logistic regression analysis of potential risk factors for seropositivity from 2016–2020 in Mongolia.

Characteristic	Number of samples	Number of positive samples	Prevalence	95% *CI		*OR (log scale)	95% *CI		p-value
				lower	upper		lower	upper	
Sex									
Female	2295	671	29.24	27.4	31.12	—	—		
Male	1397	361	25.84	23.6	28.18	1.13	0.98	1.31	0.093
Age group (years)									
0–4	649	140	21.57	18.54	24.86	—	—		
5–9	965	308	31.92	29.03	34.91	0.68	0.54	0.84	<0.001
10–14	330	109	33.03	28.12	38.24	0.65	0.49	0.87	0.003
15–19	154	51	33.12	26.05	40.81	0.65	0.45	0.94	0.022
20–24	128	17	13.28	8.24	19.96	1.62	0.97	2.87	0.077
25–29	165	58	35.15	28.17	42.64	0.61	0.43	0.88	0.006
30–34	213	55	25.82	20.3	32.00	0.84	0.59	1.19	0.3
35–39	185	46	24.86	19.06	31.45	0.87	0.6	1.27	0.5
40–44	195	46	23.59	18.05	29.91	0.91	0.64	1.33	0.6
45–49	185	48	25.95	20.04	32.6	0.83	0.58	1.21	0.3
50–54	161	51	31.68	24.87	39.14	0.68	0.48	0.99	0.039
55–59	156	40	25.64	19.28	32.9	0.84	0.57	1.26	0.4
60–64	97	27	27.84	19.66	37.31	0.77	0.49	1.25	0.3
65–69	49	15	30.61	19.1	44.34	0.7	0.39	1.33	0.3
70–75	49	17	34.69	22.54	48.59	0.62	0.35	1.14	0.11
Occupation									
Unemployed/stays at home, pensioner	1249	309	24.74	22.41	27.19	—	—		
Employed/civil servant, working in the private sector	498	115	23.09	19.55	26.94	1.07	0.85	1.36	0.6
Student/high schooler/ pupil/kindergarten	1246	409	32.83	30.26	35.47	0.75	0.64	0.89	<0.001
Herder	347	141	40.63	35.56	45.86	0.61	0.48	0.77	<0.001
Self-employed	175	41	23.43	17.62	30.11	1.06	0.74	1.53	0.8
Residence									
Arkhangai	124	90	72.58	64.27	79.85	—	—		
Bayan-Ulgii	65	13	20.00	11.7	30.9	3.63	1.94	7.25	<0.001
Bayankhongor	256	62	24.22	19.28	29.74	3.00	2.04	4.43	<0.001
Bulgan	121	17	14.05	8.73	21.06	5.17	2.97	9.45	<0.001
Darkhan-Uul	58	10	17.24	9.22	28.43	4.21	2.12	9.15	<0.001
Dornod	108	43	39.81	30.95	49.22	1.82	1.17	2.86	0.008
Dornogobi	39	3	7.69	2.21	19.13	9.44	3.29	39.9	<0.001
Dundgobi	67	9	13.43	6.86	23.08	5.4	2.68	12.1	<0.001
Gobi-Altai	303	240	79.21	74.37	83.49	0.92	0.66	1.26	0.6
Gobi-Sumber	35	6	17.14	7.49	31.97	4.23	1.83	11.6	0.002
Khentii	48	11	22.92	12.83	36.16	3.17	1.61	6.73	0.001
Khovd	251	38	15.14	11.11	19.96	4.79	3.12	7.48	<0.001
Khuvsgul	486	203	41.77	37.44	46.19	1.74	1.26	2.38	<0.001
Orkhon	101	17	16.83	10.52	25.01	4.31	2.46	7.92	<0.001
Selenge	268	46	17.16	13.01	22.02	4.23	2.81	6.44	<0.001
Sukhbaatar	25	6	24.00	10.69	42.94	3.02	1.27	8.41	0.02
Tuv	96	12	12.50	7.02	20.2	5.81	3.11	11.7	<0.001
Ulaanbaatar	671	104	15.50	12.91	18.38	4.68	3.33	6.59	<0.001
Umnugobi	52	23	44.23	31.35	57.74	1.64	0.95	2.91	0.083
Uvs	67	12	17.91	10.18	28.34	4.05	2.14	8.27	<0.001
Uvurkhangai	97	36	37.11	27.99	46.99	1.96	1.23	3.15	0.005
Zavkhan	348	31	8.91	6.25	12.24	8.15	5.21	13.00	<0.001
Season									
Fall	115	12	10.43	5.83	17	—			
Spring	2636	848	32.17	30.41	33.97	0.32	0.17	0.57	<0.001
Summer	920	167	18.15	15.76	20.74	0.57	0.30	1.02	0.079
Winter	21	5	23.81	9.72	44.57	0.44	0.15	1.49	0.2

*OR = Odds Ratio, *CI = Confidence Interval.

### Seroprevalence of tick-borne borreliosis

Among the 4724 serum samples, 162 were positive for IgG antibodies against *B*. *burgdorferi* sensu lato. Table 2 shows the seroprevalence of TBB stratified by sex, occupation, age group, and province. The seroprevalence of *Borrelia* was 3.78% (95% CI 3.1–4.5%) and 3.17% (95% CI: 2.4–4.0) in female and male participants, respectively (Table 2).

**Table 2:** Stratified seroprevalence of IgG antibodies against *Borrelia burgdorferi* sensu lato detected by ELISA in participants aged 0–75 years and the results of binary logistic regression analysis of potential risk factors for seropositivity from 2016–2020 in Mongolia.

Characteristic	Number of samples	Number of positive samples	Prevalence	95% *CI		*OR (log scale)	95% *CI		p-value
				lower	upper		lower	upper	
Sex									
Female	2858	108	3.78	3.13	4.53	—	—		
Male	1704	54	3.17	2.42	4.08	1.19	0.86	1.67	0.3
Age group (years)									
0–4	762	27	3.54	2.4	5.04	—	—		
5–9	1230	43	3.50	2.58	4.63	1.01	0.61	1.64	>0.9
10–14	428	11	2.57	1.37	4.4	1.38	0.7	2.93	0.4
15–19	195	10	5.13	2.67	8.91	0.69	0.34	1.52	0.3
20–24	143	2	1.40	0.29	4.41	2.53	0.75	15.8	0.2
25–29	214	9	4.21	2.10	7.53	0.84	0.4	1.92	0.7
30–34	255	13	5.1	2.89	8.32	0.70	0.36	1.41	0.3
35–39	226	5	2.21	0.85	4.78	1.6	0.66	4.77	0.3
40–44	234	7	2.99	1.35	5.78	1.18	0.54	2.98	0.7
45–49	220	13	5.91	3.35	9.61	0.60	0.31	1.22	0.14
50–54	206	6	2.91	1.22	5.9	1.22	0.53	3.29	0.7
55–59	191	5	2.62	1.01	5.64	1.35	0.56	4.03	0.5
60–64	116	8	6.90	3.31	12.59	0.51	0.24	1.24	0.11
65–69	64	0	0	0	0	0	0		>0.9
70–75	63	3	4.76	1.36	12.16	0.74	0.25	3.18	0.6
Occupation									
Unemployed/stays at home, pensioner	1507	51	3.38	2.56	4.39	—	—		
Employed/civil servant, works in the private sector	587	26	4.43	2.98	6.32	0.76	0.48	1.25	0.3
Student/high schooler, pupil, kindergarten	1591	64	4.02	3.14	5.07	0.84	0.58	1.22	0.4
Herder	476	12	2.52	1.39	4.23	1.34	0.73	2.66	0.4
Self-employed	211	5	2.37	0.91	5.12	1.43	0.62	4.14	0.5
Residence									
Arkhangai	210	4	1.90	0.65	4.46	—	—		
Bayan-Ulgii	78	0	0	0	0	0	0		>0.9
Bayankhongor	308	10	3.25	1.68	5.68	0.59	0.16	1.78	0.4
Bulgan	136	2	1.47	0.31	4.63	1.30	0.25	9.43	0.8
Darkhan-Uul	65	3	4.62	1.32	11.81	0.41	0.09	2.14	0.3
Dornod	146	5	3.42	1.32	7.34	0.56	0.14	2.14	0.4
Dornogobi	42	0	0	0	0	0	0		>0.9
Dundgobi	74	2	2.70	0.57	8.39	0.7	0.13	5.16	0.7
Gobi-Altai	541	2	0.37	0.08	1.18	5.15	1	37.4	0.059
Gobi-Sumber	41	0	0	0	0	0	0		>0.9
Khentii	55	4	7.27	2.5	16.37	0.26	0.06	1.14	0.064
Khovd	268	21	7.84	5.07	11.51	0.24	0.07	0.65	0.011
Khuvsgul	683	6	0.88	0.37	1.8	2.17	0.55	7.66	0.2
Orkhon	116	2	1.72	0.36	5.42	1.1	0.21	8.06	>0.9
Selenge	298	16	5.37	3.23	8.37	0.35	0.1	0.98	0.067
Sukhbaatar	31	0	0	0	0	0	0		>0.9
Tuv	105	3	2.86	0.81	7.43	0.67	0.14	3.44	0.6
Ulaanbaatar	742	33	4.45	3.14	6.11	0.43	0.13	1.09	0.11
Umnugobi	75	0	0	0	0	0	0		>0.9
Uvs	78	1	1.28	0.14	5.83	1.49	0.22	29.3	0.7
Uvurkhangai	131	2	1.53	0.32	4.81	1.25	0.24	9.09	0.8
Zavkhan	333	46	13.81	10.43	17.83	0.14	0.04	0.35	<0.001
Season									
Fall	118	9	7.63	3.84	13.46	—			
Spring	3380	104	3.08	2.53	3.7	2.48	1.14	4.76	0.012
Summer	1038	49	4.72	3.55	6.14	1.62	1.62	3.22	0.2
Winter	26	0	0	0	0	0	0	0	>0.9

*OR = Odds Ratio, *CI = Confidence Interval.

Regarding co-infections, 7 participants were positive for *Rickettsia* and *Borrelia*. All seven co-infected patients were female. The 0–4 age group accounted for one case of co-infection, 5–9 age group for three cases, 25–29 age group for one case, 45–49 age group for one case, and 55–59 age group for one case. On the basis of occupation, the co-infection was observed in two employed participants, three school children, and one herder. Two cases of co-infection were noted in Bayankhongor province, and one case each in Dornod, Dundgobi, Khovd, and Selenge provinces.

#### Age trend analysis

The seroprevalence of *Rickettsia* was nonlinearly related to age in all participants (Fig 1). From age 0–19 years, it decreased slowly (Fig 1B) with the maximum value (35.1%) observed between 25 and 29 years, followed by a gradual increase from 25.8% to 31.6% between the ages of 30 and 54 years. From age 55–64 years, it decreased slowly (Fig 1C).

To explore the potential for synergistic statistical interaction between age and *Rickettsia* seroprevalence, without arbitrarily categorising either continuous variable, generalized additive models that employ 2D smoothing curve on age and year, were used to plot a graph of the three-dimensional association (Fig 1).

The association of age and *Rickettsia* seroprevalence from the trend analysis held across all years in all participants (Fig 1B). The steeper slope of the age–seroprevalence association, relative to the year–seroprevalence association, implies, as the risk factor analysis did, that age had a stronger association with *Rickettsia* seroprevalence year. Age being the conditional variable, the association of year with *Rickettsia* seroprevalence was nearly nonlinear. Combinations of age and year that synergistically increased (or decreased) the conditional probability of TBR infection appeared on the three-dimensional risk surface.

### Risk factor analysis

To assess potential risk factors for TBR and TBB diseases, binary logistic regression analysis was performed for participants with IgG-positive serum samples. No significant association was identified for sex. The seroprevalence of *Rickettsia* was found to be 31.9% (95% CI: 29.0–34.9) among children aged 5–9 years, 33.0% (95% CI: 28.1–38.2) among those aged 10–14 years, 35.1% (95% CI: 28.1–42.6) among individuals aged 25–29 years, and 31.6% (95% CI: 24.8–39.16) among those aged 50–54 years. The binary logistic regression analysis, adjusted for the 0–4 age group, demonstrated that the risk factors were statistically significant (Table 1).

The seroprevalence of *Ricketsia* was significantly higher among herders 40.6% (95% CI: 35.5–45.8) and students 32.8% (95% CI: 30.2–35.4) than among other occupational groups.

The seasonal seroprevalence of *Rickettsia* was significantly higher in the spring season (32.1%, 95% CI: 30.4–33.9; OR: 0.3; *P* < 0.001) than in any other season (Table 1). The spring season was shown to have the highest seasonal seroprevalence of Borrelia, with an odds ratio of 2.4 (95% CI: 1.14–4.76; P < 0.012) compared to other seasons (Table 2).

The ORs of 0.61 (*P* < 0.001) for herders and 0.75 (*P* < 0.001) for students suggest that after adjusting for those who are unemployed/stay at home and are pensioners, the OR of the outcome variable was 39% lower among herders (25% lower among students) than among those who are unemployed/stay at home and are pensioners (Table 1).

Table 1 displays the findings of the binary logistic regression analysis, indicating that Bayan-Ulgii, Bayankhongor, Bulgan, and Darkhan-Uul were three to five times more likely to have a high rate of the outcome variable (with OR of 3.63, 3, 5.17, and 4.21, respectively). Similarly, Dundgobi, Dornogobi, Gobi-Sumber, Khentii, Khovd, Orkhon, Selenge, Tuv, Ulaanbaatar, Uvs, and Zavkhan had higher odds of the outcome variable than Arkhangai province, with ORs ranging from 1.74 to 9.44 and a significance level of *P*<0.02.

Binary logistic regression revealed age and study year showed a strong association with *Rickettsia* seroprevalence.

### Spatial distribution

**Fig 2:**
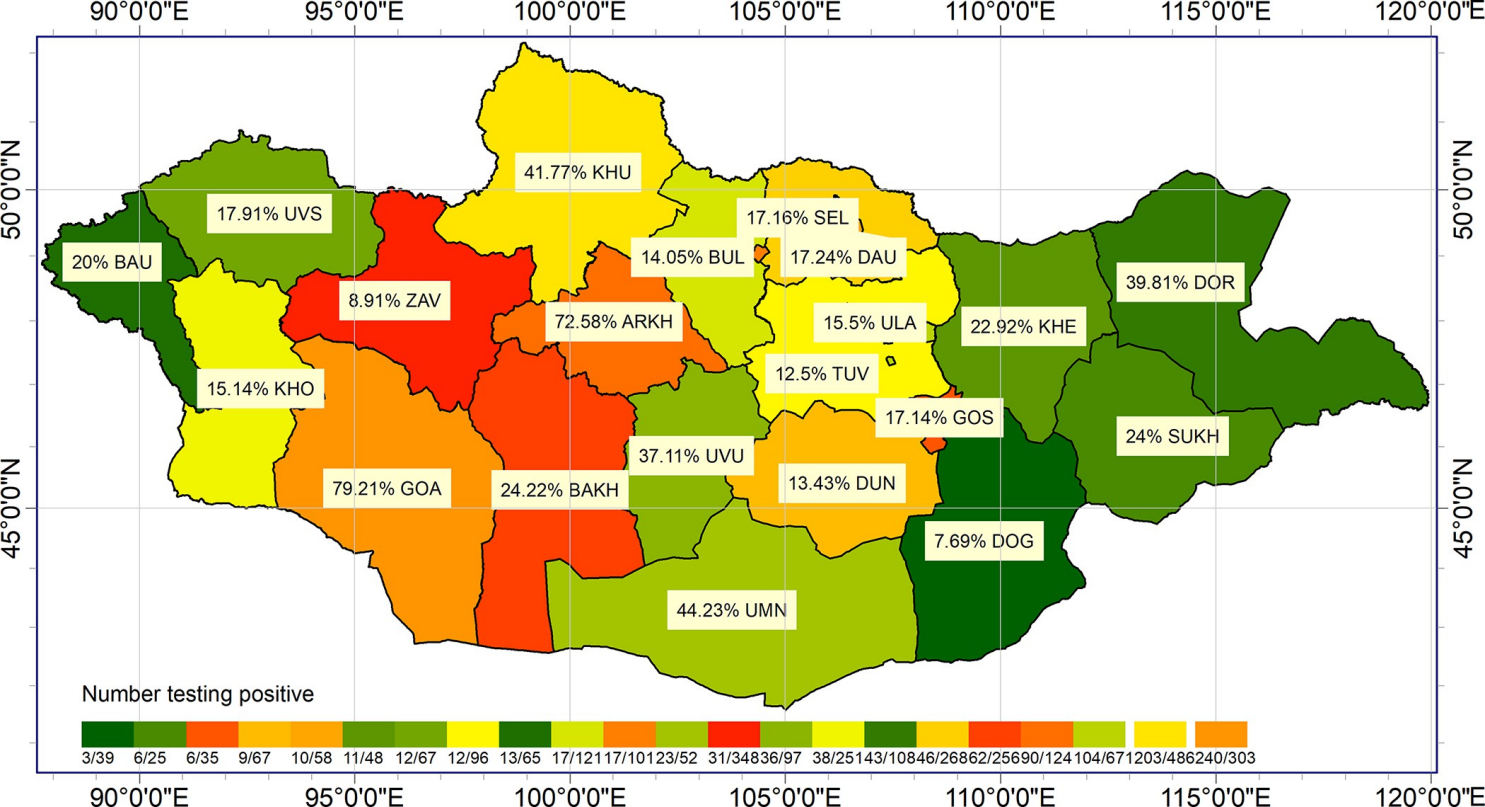
Spatial distribution of the seroprevalence of *Rickettsia* in the participants. The colour of each province corresponds to the seroprevalence of *Rickettsia* (darker colours indicate a higher seroprevalence as shown in the legend).

**Fig 3:**
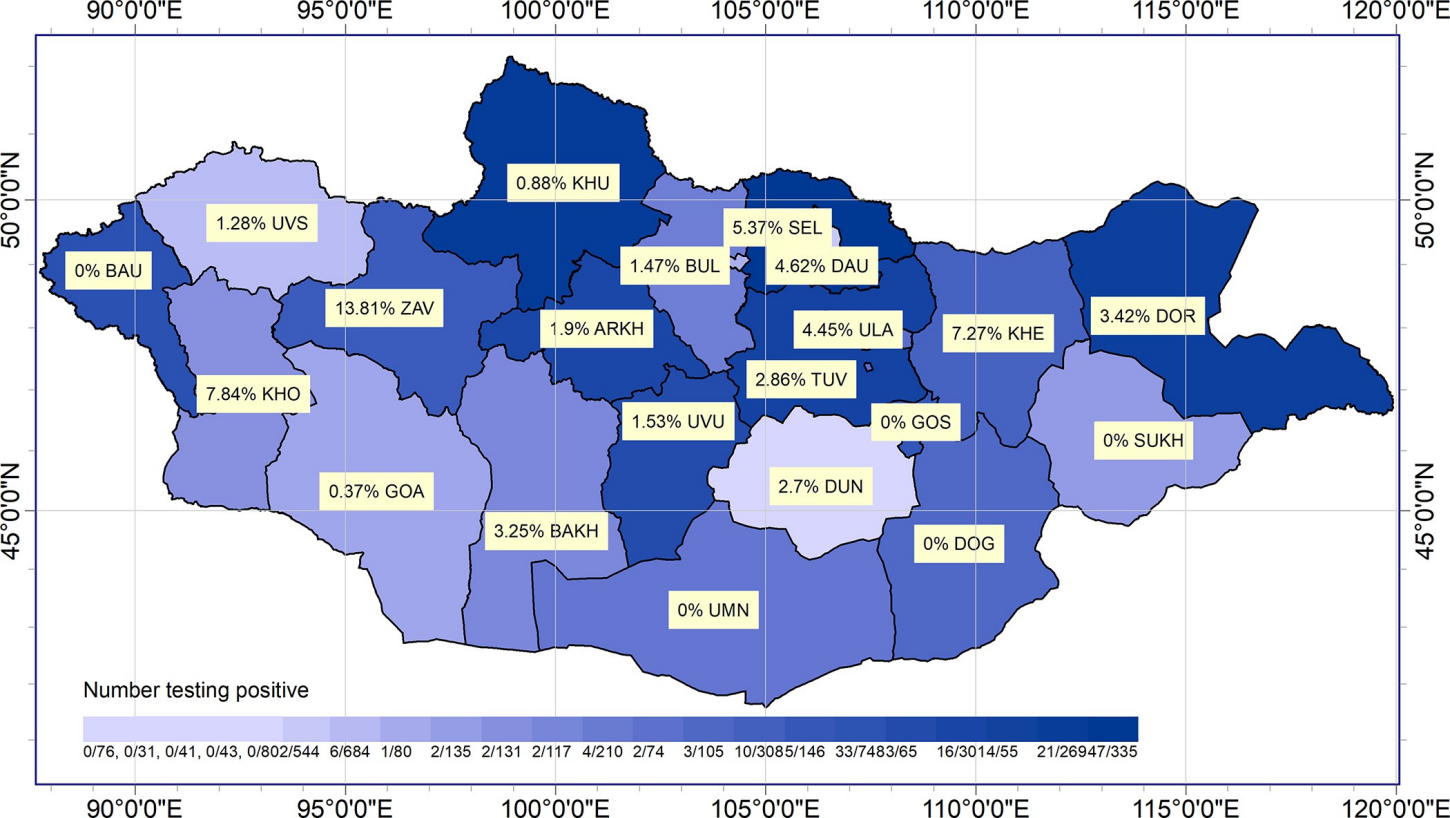
Spatial distribution of the seroprevalence of *Borrelia* in the participants. The seroprevalence evaluated for each province is mapped for *Borrelia*. The shade of each province indicates the seroprevalence of *Borrelia* (darker colours indicate a higher seroprevalence).

The seroprevalence of *Rickettsia* and TBB was mapped according to province (Figs 2 and 3, respectively). *Rickettsia* was detected in all 21 provinces, with the highest seroprevalence in the Gobi-Altai Province (79.2%) and the lowest seroprevalence in the Dornogobi Province (7.6%) (Fig 2). *Borrelia* was detected in 16 of the 21 provinces, with the highest seroprevalence in the Zavkhan Province (13.8%) and the lowest seroprevalence in the Uvs Province (1.2%) (Fig 3). The geographic distribution ofTBR and TBB differed, suggesting a difference in their areas of endemicity. The seroprevalence of both *Rickettsia* and *Borrelia* was 10–15% higher in western provinces than in the eastern provinces.

## Discussion

In this study, 4724 sera from a wide geographical range in Mongolia were analysedfor *Rickettsia* and *Borrelia* seroprevalence.We observed a high number of tick-borne infections due to the *Rickettsia* and *Borrelia* epidemics that swept through Mongolia, peaking between 2016 and 2020.

Von Fricken et al. [14] previously reported a high seroprevalence of *Borrelia* antibodies in Zavkhan (25%) and Selenge (20%) Provinces after the 2007–2017 *Borrelia* epidemic in Mongolia. In this study, the seroprevalence of IgG antibodies against *B*. *burgdorferi* sensu lato was 13.8% and 5.3% in Zavkhan and Selenge Provinces, respectively.

In a previous study, the seroprevalence of *Rickettsia* in Mongolia was concentrated in the 0–18-year (21%), 19–49-year (28%), and 40–49-year (33%) age groups [3]. However, in this study, we observed that the seroprevalence of *Rickettsia* was higher in the 25–29-year age group than in the other age groups. This increase may be explained by the fact that in rural areas of Mongolia, 25 to 29-year-olds spend more time outdoors and are more likely to engage in outdoor activities such as herding their domestic animals in grasslands, camping, gardening, and horse-riding than other age groups. Furthermore, the seroprevalence of *Rickettsia* was higher in the 5–9- and 10–14-year age groups than in the 35–39- and 40–44-year age groups. It is possible that young children cannot adequately protect themselves from tick bites or infestation. Although the usefulness of conducting inspections of the body for ticks has not been confirmed, there is evidence that removal of *Ixodes scapularis* within 36 hours after attachment will reduce the risk of TBB [19,20]. Contrarily,Steiner-Khamsi and Gerelmaa foundthat about 40% of herder families wantedtheir children to liveinschooldormitories. Afterthe first school year, many herder families withdrew their children because they performed poorly or because theywere required to repeat a grade due to an poor health dormitory [21]. More girls than boys from herder families are admitted to school. The imbalance is greatest at the upper secondary level, where 60% of girls are admitted to school. Accordingtoan earlier report on male child labour in agriculture and animal husbandry, the majority of male children in rural areas never attend school or drop out [22]. Sincetheyaremore likely to be outside and handle animals, children from herder families maybe more susceptible to TBDs.

*Rickettsia* and *Borrelia* serprevalence was higher among herders and students than among those with other occupations. Mongolian herders herd their livestock in common open pastures, whereas nomadic or semi-nomadic herders move from one grazing area to another with their livestock [23].Herders, as a professional group, work in different ecological areas, and their surroundings are a natural ecosystem for ticks. Sex differences in seroprevalence may be attributed to lifestyle or could be attributed to sampling bias if women have a higher tendency to seek medical attention in hospitals than men [24–26]. Tick checks are alsoassociatedwith higher levels of knowledge, according to researchers from The Netherlands [27]. The children ofMongolian livestock herders help their parents raise their young domestic animals. Mongolia is one of the few countries in which nomadic and semi-nomadic lifestyles still exist [3,23]. Consequently, Mongolian herder families, especially children, live in close proximity with domestic animals; therefore, TBDs and zoonoses are major public health problems in this population [1,3–5]. Herders’ children are exposed to tick bites when working with wool and cashmere, wool-baiting, combing goats, and shearing animals.

A study in Mongolia assessed the association between symptoms and tick bites based on serological evidence of *Rickettsia* and *Borrelia* infection; however, as the study was conducted in a hospital setting, a selection bias potentially existed because participation was limited to those who presented to the hospital for treatment, and the nomadic herder population may have been underrepresented [14].The prevention of TBDs in Mongolia largely depends on the beliefs, practices, and awareness of members of the herder community. Nomadic and semi-nomadic herders with less than 100 domestic animals are often isolated [23] because they herd in open spaces, and it is difficult for them to access public services, especially healthcare and education. This might explain the large difference between experiencing symptoms of disease and seeking treatment [28]. Not only do Mongolian [3,4] herders have a high seroprevalence of TBR and TBB, herders in China [29], Ghana [30], North Cameroon [31], and Jordan [32] also have a higher seroprevalence of *Rickettsia* and *Borrelia* than those in other occupations. According to studies in Europe, the seroprevalence of *B*. *burgdorferi* sensu lato is higher in agricultural, farming, and forest industry workers than in the general population [2,6].

Tick-borne diseases are prevalent in the human population and may become an important and persistent threat to human health [8]. Humans are occasional hosts of ticks but do not play a role in maintaining tick-borne agents in nature [33]. In one study, spotted fever group *Rickettsia* was detected in 19.5% (73/374) of humans [3] and 20.4% (478/2342) of livestock [4] in Mongolia. TBDs can directly affect the lives of Mongolians by causing illness in humans and, indirectly, can cause economic losses due to zoonoses in livestock. Therefore, numerous epidemiological studies have been conducted to determine the seroprevalence of antibodies against the *Rickettsia* spotted fever group [4,34,35], *B*. *burgdorferi* sensu lato [3,5,36,37], *Anaplasma* spp. [38,39], and molecular identification of tick-borne encephalitis virus [40,41] in Mongolia; however, most previous studies were limited by small sample sizes and limited geographic coverage.

In this study of a hospital-based population between 2016 and 2020, the seropositivities of *Rickettsia* in all provinces and *Borrelia* in 16 provinces were 21.8% and 3.4%, respectively.However, previous studies (with sample sizes of 316, 374, 335, and 150) might not have been adequately facilitated [3–5,14]. It is often difficult to compare field surveys on TBR and TBB because of varying sampling methods and sample sizes. Our study confirmed a higher seroprevalence of *Rickettsia* than that of *Borrelia*. In one study, all of the *Dermacentor* spp. ticks tested in Mongolia were positive for *Rickettsia* [33,42]. In another study, *Dermacentor nuttalli* was detected in all the ecological areas and provinces of Mongolia [15]. *Ixodes persulcatus* ticks are the known vectors of borreliosis in Mongolia, whereas *Dermacentor* and *Haemaphysalis* spp.ticks are vectors carriers of *Rickettsia* [43]. Differences in biological niches of ticks from northern to southern provinces may partially explain the ecological and geographical differences in the seroprevalence of *Rickettsia* and *Borrelia*.The burden of TBDs in the Selenge Province has been reported previously [5]; however, in this study, we detected a high seroprevalence of *Borrelia* in the Zavkhan Province. The high seroprevalence of *Borrelia* in Zavkhan Province is of concern because of the high volume of native and foreign visitors who pass through the province. We infer that the lower seroprevalence of Rickettsia and *Borrelia* among humans in the Gobi-Altai and Gobi-Sumber provinces, which are situated adjacent to the desert, is attributable to decreased grazing.

According to our study, *Rickettsia* seroprevalence was higher in the western provinces than in the eastern provinces. Altantogtoh et al. [44] previously reported that *Rickettsia* was more prevalent in tick pools in the western and eastern provinces of Mongolia than in those in the other provinces. This shows that there is considerable geographic variability in the ecology of *Rickettsia* spp. pathogens. These differences may reflect differences in human contact with ticks and infected vectors, but more research is needed to clarify these differences.

In previous studies, co-infection with *Borrelia burgdorferi* and *Anaplasma phagocytophylilum* was found [45]. Moreover, this was detected with *Babesia caballi* and *Babesia equi* [46] infections, according to the relative frequency of pathogen detection in different ticks, as described earlier for *I*. *persulcatus* in Mongolia [45,46]. In addition, co-infection with *Borrelia* and *Babesia* was found, including infection with *D*. *nuttali* (1.1%), in *I*. *persulcatus* (28.6%), and *H*. *asiaticum* (2.3%) [15]. In this study, *Rickettsia* and *Borrelia* co-infection was found in humans (0.14%). These findings emphasize the importance of preventing tick bites and monitoring for multiple infections in both ticks and humans.

The strengths of this study include the large sample size comprising a population with a wide age range, the sensitivity and specificity of the diagnostic tests used to measure anti-*Rickettsia* and *Borrelia* antibodies, and the robust analysis of age trends and risk factors. Nevertheless, this study had some limitations. First, the 4724 participants were patients who sought treatment at health facilities; therefore, we were unable to determine the seroprevalence of asymptomatic infection. Collectively, this findings demonstrate the importance of further research in this area to gain a better understanding of this situation.Second, this study did not investigate participants’ knowledge, attitudes, beliefs, and practices with regard to TBDs. Third, the study focused on enrolment at hospitals when nomadic movement could lead to exposure elsewhere, especially given the prolonged period for which antibodies can be detected.

In summary, this study determined the seropositivity of *Rickettsia* and *Borrelia* and the risk factors for infection among children and adults in a hospital-based population from Mongolia.The seroprevalence of *Rickettsia* was high, especially in herders, and seroprevalence studies on *Rickettsia* and *Borrelia* should be considered for inclusion in the future. *Rickettsia* was found in all provinces, whereas *Borrelia* was found in only 16 provinces of Mongolia. Furthermore, we found that occupation and age were risk factors for rickettsial infections.

## Supporting information

S1 File Information sheet for participant. (DOCX)

S2 File Written informed participant consent. (DOCX)

S3 File Information sheet for parents/guardians (Below 12 years old). (DOCX)

S4 File Written informed parental consent (Below 12 years old). (DOCX)

S5 File Information sheet for children age 12–17 years old. (DOCX)

S6 File Assent form (english version). (DOCX)

S7 File Protocol of Anti-Borrelia ELISA (IgG). (DOCX)

S8 File Spotted fever rickettsia IgG. (DOCX)
