# Supplementary material for: Seroprevalence and risk factors of Borrelia burgdorferi sensu lato and Rickettsia species infection in humans in Mongolia, 2016–2020
Source: PLoS One. 2023 Aug 8;18(8):e0289274. doi: 10.1371/journal.pone.0289274 (PMC10409273; doi:10.1371/journal.pone.0289274)
Supplement: S2 File — (DOCX) [file pone.0289274.s002.docx]

**ANNEX 2**

**Seroprevalence and risk factors of *Borrelia burgdorferi* sensu lato and *Rickettsia* species infection in humans in Mongolia, 2016–2020**

**WRITTEN INFORMED Participant CONSENT**

**(ENGLISH VERSION)**

I was given the opportunity to read this information sheet. Its contents were explained and discussed with me. I was also given the chance to ask questions and I am happy with the answers I received.

_______________________ __________________________

Name of patient Name of Person Giving Consent

______________________ ___________________________ __________

Name of Person Obtaining Consent Signature of Person Obtaining Consent Date of Signing

______________________

*(Mm/dd/yy)*

**WITNESS (IF NEEDED)**

This is to confirm that the information given to the potential participant above was found in the information sheet. I also heard the purpose of the survey and its procedures, the benefits and the risk it will do were discussed and explained. S/He was also given the opportunity to ask questions. S/He was also informed that s/he does not have to join the survey if s/he does not like to and that s/he can stop the interview at any time. I also witnessed the person gave her/his verbal consent to participate in the survey.

_______________ _______________________ _____________________

Name of Witness Signature of Witness Date of Signing

*(mm/dd/yy)*
