# Supplementary material for: Seroprevalence and risk factors of Borrelia burgdorferi sensu lato and Rickettsia species infection in humans in Mongolia, 2016–2020
Source: PLoS One. 2023 Aug 8;18(8):e0289274. doi: 10.1371/journal.pone.0289274 (PMC10409273; doi:10.1371/journal.pone.0289274)
Supplement: S3 File — (DOCX) [file pone.0289274.s003.docx]

**ANNEX 3**

**Seroprevalence and risk factors of *Borrelia burgdorferi* sensu lato and *Rickettsia* species infection in humans in Mongolia, 2016–2020**

**INFORMATION SHEET FOR PARENTS/GUARDIANS**

ENGLISH VERSION

(Below 12 years old)

**Background and Purpose of the survey**:

Tick-borne diseases are a growing public health concern in Mongolia and a cause of significant disease burden in humans because ticks serve as vectors in the transmission of pathogens. Tick-borne borreliosis (also known as Lyme disease) is caused by the spirochete *Borrelia burgdorferi* sensu lato, whereas tick-borne rickettsiosis is caused by a gram-negative intracellular bacterium. Tick-borne borreliosis and tick-borne rickettsiosis constitute the worldwide Tick-borne diseases s with clinical manifestations. Tick-borne borreliosis causes several neurological and arthritic symptoms, such as headache, paralysis, and erythema migrans. Tick-borne rickettsiosis usually manifests as mild fever, muscle aches, rash, cough, and nausea.

The purpose of the research is to investigate the presence of tick-borne diseases in humans.

**Procedure:**

First, I will give you some information about tick-borne diseases. Tick populations are increasing, and their geographic ranges are expanding, as are suitable habitats for these arthropod vectors and the pathogens that they carry. Ticks can transmit bacterial, parasitic, and viral pathogens and often harbor more than one agent simultaneously.

Second, if you consent to your child/children's participation in this survey, he/she will be asked to take a blood test. A small amount of blood, equal to about a teaspoon, will be taken from his/her arm with a syringe. This blood will be tested for the presence of tick-borne diseases in Ulaanbaatar city. Results of the blood test performed will be given to the Province Health Department. At the end of the research, in one year, any left over blood sample will be destroyed.

Your child also will be requested to answer questions about his/her. Finally, if your child has an infection of tick borne disease, we will give you an advise on the future treatments.

**Benefits to the Child participant**:

If your child will participate in this research, s/he will have the following benefit:

- his/her disease will be diagnosed at no charge to you

Participation of your child for this research helps us to decrease the prevalence of tick-borne diseases in your province.

**Risk from survey procedure**:

There are no clear risks associated with blood tests.

**Voluntary Participation and Confidentiality of information**:

Your decision to have your child participate in this study is entirely voluntary. It is your choice whether to have your child participate or not. You may also choose to change your mind later and stop participating, even if you agreed earlier, that is no problem.

The information that we collect from this research project will be kept confidential. Information about your child that will be collected during the research will be put away and no one but the researchers will be able to see it. Any information about your child will have a number on it instead of his/her name. Only the researchers will know what your child number is and we will lock that information up with a lock and key. It will not be shared with or given to anyone except researchers.

Data files containing personal information will be stored in a locked computer with password protection at the Mongolian National University of Medical Sciences in Ulaanbaatar city. Access to data files will be limited to the researchers only**.** At the end of the study, data files containing personal identifiers will either be destroyed. Your child will not be personally identified when research results are published or discussed at seminars, conferences or in any other format.

**Contact Information: If you have any questions about the study,** you can contact the following persons:

| Dr. Narankhajid Myadagsuren | Department of Biology and Mongolian National University of Medical Sciences, Ulaanbaatar city | Tel: 976 -99132428 |
| --- | --- | --- |
| M.D. Dashdavaa Ganbold | Department of Biology and Mongolian National University of Medical Sciences, Ulaanbaatar city | Tel: 976-91119589 |
