# Supplementary material for: Seroprevalence and risk factors of Borrelia burgdorferi sensu lato and Rickettsia species infection in humans in Mongolia, 2016–2020
Source: PLoS One. 2023 Aug 8;18(8):e0289274. doi: 10.1371/journal.pone.0289274 (PMC10409273; doi:10.1371/journal.pone.0289274)
Supplement: S6 File — (DOCX) [file pone.0289274.s006.docx]

**ANNEX 6**

**Seroprevalence and risk factors of *Borrelia burgdorferi* sensu lato and *Rickettsia* species infection in humans in Mongolia, 2016–2020**

**ASSENT FORM (ENGLISH VERSION)**

(12-17 years old)

The above information has been discussed with me in detail and has been understood by me. I was given opportunity to ask questions and was satisfied with answers given. My participation in this project is voluntary and I can withdraw anytime without prejudice to any medical treatment that I ought to receive. My signature below signifies my consent to participate in this study.

_______________________ __________________________

Name of Child Signature of Child

___________________

Date of signing *(mm/dd/yy)*

___________________ __________________________

Name of Person Obtaining Consent Signature of Person Obtaining Consent

Date of signing *(mm/dd/yy)*
