# Supplementary material for: Seroprevalence and risk factors of Borrelia burgdorferi sensu lato and Rickettsia species infection in humans in Mongolia, 2016–2020
Source: PLoS One. 2023 Aug 8;18(8):e0289274. doi: 10.1371/journal.pone.0289274 (PMC10409273; doi:10.1371/journal.pone.0289274)
Supplement: S7 File — (DOCX) [file pone.0289274.s007.docx]

**Anti-Borrelia ELISA (IgG)**

**Procedure**

|  |  |
| --- | --- |
| Sample incubation:  (1st step) | Transfer 100 µl of the diluted serum samples. One serum sample per well. Incubate at room temperature (+18^0^C to +25^0^C) for 30 minutes. |
| Washing: | Aspirate off the liquid from each well and wash 3 times each with 450µl working-strength universal buffer on a rocking shaker. Leave the wash buffer in each well for 30 to 60 seconds per washing cycle, and then empty the wells. |
| Conjugate incubation:  (2nd step) | Pipette 100 µl ready for use diluted enzyme conjugate (peroxidase-labelled anti-human-IgG) into each of the microplate wells. Incubate for 30 minutes at room temperature (+18^0^C to +25^0^C). |
| Washing: | Aspirate off the liquid from each well. Wash as described above. |
|  |  |
| Substrate incubation:  (3 rd step) | Pipette 100µl chromogen/substrate solution into the channels of the incubation tray. Incubate for 15 minutes at room temperature (+18^0^C to +25^0^C), protect from sunlight. |
| Stopping: | Aspirate off the liquid from each well and pipette 100µl of stop solution into of the microplate wells in the same order and same speed as the chromogen/substrate solution was introduced. |
| Evaluation: | Results should be read once strips have dried. |

**Measurement**

Adjust the ELISA Microwell Plate Reader to **zero** using the **substrate blank in well A1**.

If - due to technical reasons - the ELISA reader cannot be adjusted to zero using the substrate blank in well A1, subtract the absorbance value of well A1 from all other absorbance values measured in order to obtain reliable results!

**Measure the absorbance** of all wells at **450 nm** and record the absorbance values for each control and patient sample in the distribution and identification plan.

*Dual wavelength reading using 620 nm as reference wavelength is recommended.*

Where applicable calculate the **mean absorbance** **values** of all duplicates.

**Run Validation Criteria**

In order for an assay to be considered valid, the following criteria must be met:

- Negative Control < 0.2 absorbance units
- Positive Control > 0.8 absorbance units
- Cutoff Calibrator 0.25-0.55 absorbance units
- Cutoff Calibrator / Negative Control ratio > 1.5
- Positive Control / Cutoff Calibrator ratio > 2.0

**Calculation of Results**

The cut-off is the mean absorbance value of the Cut-off control determinations.

*Example*: Absorbance value Cut-off control 0.54 + absorbance value Cut-off control 0.52 =1.06 / 2 = 0.53

Cut-off = 0.53

**Interpretation of Results**

Samples are considered POSITIVE if the absorbance value is higher than 10% over the cut-off.

Samples with an absorbance value of 10% above or below the cut-off should not be considered as clearly positive or negative

- **grey zone**

It is recommended to repeat the test again 2 - 4 weeks later with a fresh sample. If results in the second test are again in the grey zone the sample has to be considered **NEGATIVE**.

Samples are considered **NEGATIVE** if the absorbance value is lower than 10% below the cut-off.

**Results in Nova Tec Units**

*Example:* $\frac{1.786 x 10}{0.53}= 34 NTU (NovaTec Units)$

Cut-off: 10 NTU

Grey zone: 9-11 NTU

Negative: <9 NTU

Positive: >11 NTU
